# A pH-responsive dual-drug nanoplatform for stromal remodeling and enhanced chemotherapy via MMP3/TGF-*β* inhibition

**DOI:** 10.1016/j.ijpx.2026.100489

**Published:** 2026-01-13

**Authors:** Tao Tan, Yihan Wang, Ran Cheng, Dongsheng Yang

**Affiliations:** a College of Life Science, Zhuhai College of Science and Technology, Zhuhai 519041, PR China; b College of Life Science, Jilin University, Changchun 130012, PR China

**Keywords:** Quercetin, cancer-associated fibroblasts, Matrix metalloproteinase 3, Stromal remodeling, Enhanced chemotherapy

## Abstract

The dense, fibrotic extracellular matrix (ECM) generated by cancer-associated fibroblasts (CAFs) within the tumor microenvironment (TME) presents a formidable physical barrier that severely limits the penetration and efficacy of chemotherapeutic agents. This study aimed to design and validate a pH-responsive, dual-drug nanosystem (RPAE-QM) capable of overcoming this stromal resistance by coordinated delivery of a stromal-modulating agent and a potent cytotoxic payload. The RPAE-QM nanosystem was constructed to co-encapsulate the stromal-modulating agent quercetin (Que) and the chemotherapeutic drug DM1. RPAE-QM exhibited significant pH-responsive drug release, leading to strong synergistic cytotoxicity and superior tumor destruction capability in 3D spheroid models. Mechanistic investigation provided a definitive explanation for this efficacy. Molecular docking and molecular dynamics simulations predicted that Que. has high affinity and exceptional kinetic stability when binding to MMP-3. Subsequent experiments confirmed the downstream consequences of this interaction: treatment with Que. caused dose-dependent inhibition of both MMP-3 and TGF-*β*_1_ secretion from CAFs. Moreover, this was accompanied by a significant, concentration-dependent reduction in the phosphorylation of Smad2/3, a key downstream effector of the TGF-*β* signaling pathway. The RPAE-QM nanosystem provides an effective dual-action strategy, simultaneously addressing the stromal barrier while delivering a potent cytotoxic agent. Mechanistically, our findings indicate that Que. suppresses the pro-fibrotic MMP3/TGF-*β*/Smad signaling axis in our CAF model. This work therefore introduces a dual-action therapeutic concept, offering a mechanistically-defined approach to disrupt stromal barriers and improve drug efficacy at the pre-clinical proof-of-concept stage.

## 1 Introduction

Nanotechnology offers a powerful platform for targeted cancer therapy, leading to clinically approved nanosystems such as Doxil®, Abraxane®, and Onivyde® (Lin and Jiang, 2025; Wang et al., 2024). However, their clinical success has often been modest, a limitation largely attributed to poor penetration into the dense tumor mass (Tan et al., 2019). This challenge is primarily imposed by the tumor microenvironment (TME) —a complex network of disorganized vasculature, stromal cells, and a dense extracellular matrix (ECM) (Wang et al., 2023; De Visser and Joyce, 2023). Within the TME, cancer-associated fibroblasts (CAFs) are the principal architects of this therapeutic barrier. By excessively secreting ECM components like collagen and fibronectin, CAFs construct a stiff, fibrotic stroma that not only promotes tumor progression and invasion but also physically impedes the diffusion of nanomedicines (Liang et al., 2023a; Chhabra and Weeraratna, 2023). This formidable biological barrier effectively traps drugs at the tumor periphery, leading to suboptimal distribution and poor clinical outcomes (Li et al., 2024). Therefore, strategies that move beyond merely targeting cancer cells to simultaneously reprogram CAFs are urgently needed and highly promising for the treatment of CAF-rich solid tumors like breast, pancreatic, and colorectal cancers.

In direct response to this need, modulating CAFs to dismantle the fibrotic barrier has emerged as a highly promising strategy. Among various candidate agents, the naturally occurring bioflavonoid, Quercetin (Que), has attracted considerable attention for its ability to effectively reprogram activated CAFs, downregulating their expression of collagen and fibronectin, thereby “normalizing” the TME to enhance drug penetration (Duan et al., 2022; Ge et al., 2023). Beyond its matrix-remodeling capabilities, Que. exhibits remarkable pleiotropic anti-cancer effects, functioning as a potent chemosensitizer that potentiates the efficacy of conventional drugs like doxorubicin and cisplatin (Zhao et al., 2023; Meng et al., 2024). These multifaceted properties make Que. an almost ideal adjuvant agent for combination cancer therapy. However, the clinical translation of free Que. is severely hampered by its exceedingly poor aqueous solubility, low bioavailability, and non-specific biodistribution, which prevent it from reaching therapeutic concentrations at the tumor site (Cai et al., 2013).

Nanoparticle delivery systems offer a direct and powerful solution to precisely these pharmaceutical limitations (Sun et al., 2023; Nguyen et al., 2024). By encapsulating Que. within a nanocarrier, its aqueous solubility can be dramatically improved, its degradation in circulation can be prevented, and its accumulation in tumor tissue can be significantly enhanced through both passive (the EPR effect) and active (e.g., RGD-peptide functionalization) targeting strategies (Yang et al., 2024). This immediately suggests a compelling therapeutic hypothesis: a nanosystem designed to deliver Que. could act as a “path-opener”. Upon accumulation in the TME, such a nanosystem would release Que. to remodel the stroma and “soften” the tumor, thereby paving the way for a co-delivered or subsequently administered cytotoxic agent to achieve deeper penetration and greater therapeutic efficacy. The rational design of such a Que-based nanoplatform, therefore, holds immense potential to increase the effectiveness of chemotherapy for solid tumors.

However, a critical barrier to achieving true “rational design” persists: the precise molecular mechanism by which Que. regulates CAFs remains largely uncharacterized. While its phenomenological effects on collagen reduction are recognized, the absence of a defined molecular target hinders the development of truly optimized systems and prevents a full understanding of its therapeutic action. To move beyond simple encapsulation and engineer a truly intelligent nanomedicine, it is essential to first understand how the active agent works at a fundamental level. To address this knowledge gap, we turned to computational methods. Advances in computer-aided drug design, including virtual screening and expanding protein structure databases, have revolutionized the exploration of targets for natural compounds (Ouyang et al., 2023). We hypothesized that by employing a comprehensive strategy of database mining combined with molecular docking and molecular dynamics simulations, we could uncover the direct molecular target of Que. in CAFs. This mechanistic investigation is not merely an academic exercise; it is an essential prerequisite for validating and rationally optimizing the design of our therapeutic platform.

To address this challenge, we designed and validated a multi-pronged therapeutic strategy embodied in a single, pH-sensitive nanosystem (RPAE-QM). This system co-delivers the potent microtubule inhibitor maytansine (DM1) as the cytotoxic payload, and Que. as a stromal-reprogramming agent (Shame 1). The nanosystem is constructed from an amphiphilic block copolymer engineered with two key features: (1) Active Targeting: Its surface is decorated with a CRGDfK peptide to target *αvβ*_3/5_ integrins, which are overexpressed on tumor vasculature and breast cancer cells, ensuring preferential accumulation at the tumor site (Xu et al., 2025). (2) Smart Release: Its poly (β-amino ester) (PAE) core acts as a precise environmental sensor (Tan et al., 2024). While stable in systemic circulation (pH 7.4), the PAE backbone protonates in the acidic TME (pH ∼6.5), triggering rapid structural disassembly and the synchronized release of both DM1 and Que. Our central hypothesis is that co-delivery of these two agents enables a synergistic anti-tumor effect. Que. is hypothesized to reprogram CAFs and mitigate the restrictive ECM barrier, thereby enhancing the penetration and cytotoxic efficacy of the co-delivered DM1. Through a comprehensive set of in vitro assays using 2D and 3D models, we demonstrate the validity of this dual-action approach. Furthermore, we elucidate the specific molecular mechanism by which Que. regulates CAFs—by suppressing the MMP-3/TGF-*β*/Smad signaling axis. This study describes the design and in vitro evaluation of a dual-action nanosystem engineered to address CAF-induced therapeutic resistance. We propose a promising strategy for simultaneously targeting cancer cells and stromal fibroblasts, offering a mechanistically-driven approach to potentially enhance drug efficacy within dense, fibrotic tumor models.

## 2 Material and methods

### 2.1 Materials

Quercetin (Que), mertansine (DM1), methylene blue (MB), 5,5’-Dithiobis- (2-nitrobenzoic acid) (DTNB), and L-glutathione (GSH) were obtained from Shanghai Macklin Biochemical Co. Ltd.. 1,1′-dioctadecyl-3,3,3′,3′-tetramethylindodicarbocyanine (DiD), 2′,7’-Dichlorodihydrofluorescein diacetate (DCFH-DA) and Goat anti rabbit IgG (Alexa Fluor® 488) were purchased from Shanghai Beyotime Biotechnology Co. Ltd.. DSPE-PEG_2K_ were supplied by AVT Pharmaceutical Tech Co., Ltd. (Shanghai, China). The pH-sensitive amphiphilic block polymers PAE-PEG_5K_-RGD and PAE-PEG_5K_ were purchased from Xi'an Ruixi Biological Technology Co. Ltd. RPMI 1640 medium, Dulbecco's modified Eagle's medium (DMEM), fetal bovine serum (FBS), and calf bovine serum (CBS) were purchased from GIBCO (CA, USA).

### 2.2 Cell lines

Murine metastatic 4 T1 breast cancer cells and mouse NIH3T3 embryonic fibroblast cells were obtained from the Shanghai Cell Bank, Chinese Academy of Sciences (CAS). The 4 T1 and NIH3T3 cells were respectively cultured in the RPMI 1640 medium supplemented with 10% FBS and DMEM supplemented with 10% CBS, 100 U/mL of penicillin, and 100 μg/mL of streptomycin at 37 °C and 5% CO_2_ in a humidified incubator. Meanwhile NIH3T3 cells were pre-activated with 10 ng/mL TGF-*β* to differentiate into CAFs.

### 2.3 Preparation and characterization of Que. and DM1 coloaded nanoparticles (RPAE-QM)

The RPAE-QM was prepared by the rotary-film evaporation method with Que., DM1, DSPE-PEG_2k_, and PAE-PEG_5k_-RGD (weight ratio: 1:0.1:2:10). Briefly, Que., DM1, DSPE-PEG_2k_, and PAE-PEG_5k_-RGD were dissolved in a chloroform/methanol (*v*/v = 1:1) solution in a round bottom flask and rotary evaporated under reduced pressure to dryness to form a thin film. Then, the film was dispersed in phosphate buffer solution (PBS, pH 7.4) and sonicated with a probe for 5 min. Finally, free drugs were removed through a microporous filter (0.22 μm) to obtain the Que. and DM1 coloaded nanoparticles (RPAE-QM). In contrast, counterpart formulations of PAE-QM containing PAE-PEG_5k_ instead of PAE-PEG_5k_-RGD, the Que-loaded nanoparticles (RPAE-Q) and DM1-loaded nanoparticles (RPAE-M) were prepared using the same procedure. In addition, the DiD-loaded nanoparticles (RPAE-QMD, PAE-QMD, RPAE-QD, RPAE-D and PAE-D) were also prepared using the same procedure, but the amount of DiD add was five times that of DM1.

The particle size distribution and ξ potential of RPAE-QM in PBS (pH 7.4) or PBS (pH 6.5) were measured using dynamic light scattering (DLS; Nanoplus-3, Micromeritics). The morphology of RPAE-QM in PBS (pH 7.4) and PBS (pH 6.5) was determined using transmission electron microscopy (TEM, Tecnai G2 F20 S-Twin, FEI) after negative staining with uranyl acetate.

The encapsulation efficiency (EE) of Que. and DM1 in the nanosystem (RPAE-Q, RPAE-M, RPAE-QM, PAE-QM) was measured. Unentrapped Que. was separated from these nanoformulations by centrifuging at 3000 rpm for 5 min. The Que. content in the supernatant (Que entrapped in the nanoformulation) was determined using UV–vis absorption spectrum analysis (Epoch, Biotek, USA) at a detection wavelength of 370 nm. Unentrapped DM1 was separated from these nanoformulations by ultrafiltration centrifugation (50,000 Da, ultrafiltration tube). The amount of DM1 entrapped was analyzed using using a high-performance liquid chromatography (HPLC) system (Waters Alliance, USA) equipped with an Agilent ZORBAX SB-C18 column (5 μm, 4.6 × 250 mm) under the following conditions: flow rate, 1.0 mL/min; detection wavelength, 254 nm. The mobile phase consisted of 50% acetonitrile with 0.1% tri-fluoroacetic acid and 50% water with 0.1% tri-fluoroacetic acid. The encapsulation efficiency (EE) was determined as the ratio of the amount of encapsulated drug to the total amount in the formulation. The measurements were performed in triplicate.

To evaluate the pH-responsive drug release profiles, RPAE-QM was incubated with PBS at pH 7.4 and pH 6.5, respectively, and then incubated at 37 °C for 24 h. The accumulated amounts of Que. and DM1 released at certain time intervals were analyzed using the aforementioned method. To evaluate the stability over time, RPAE-QM were incubated with PBS (pH 7.4) and PBS (pH 7.4) containing 10% fetal bovine serum for 72 h. The EE of Que. and DM1 in the RPAE-QM and particle size of RPAE-QM at certain time intervals of incubation was successively determined by the above-mentioned method. The measurements were performed in triplicate.

### 2.4 Cellular uptake

To test the uptake of the nanosystems (RPAE-QM and PAE-QM), the DiD loaded nanoparticles (RPAE-QMD and PAE-QMD) were prepared. Free DiD,RPAE-QMD and PAE-QMD at pH 7.4 or pH 6.5 in 4 T1 cells was visualized under an inverted fluorescence microscope (IX73, Olympus, Japan). The 4 T1 cells were seeded in a 24-well plate placed with sterile round glass coverslips at 1 × 10^4^ cells/well. After 24 h, the nanosystem was added to each well at 1 μg/mL DiD and incubated for 4 h. Then, the cells were stained with Hoechst33342 (Blue, Beyotime) for visualization under an inverted fluorescence microscope. Meanwhile, the cellular uptake of the nanosystem was also quantified by flow cytometry (CytoFlex, Beckman, USA). Cells (4 T1 or NIH3T3) were seeded into 12-well culture plates at 2 × 10^5^ cells per well, cultured overnight, and then incubated with DiD, RPAE-QMD and PAE-QMD at 1 μg/mL of DiD at pH 7.4 or pH 6.5. Cells treated with saline were used as negative controls. After 4 h, the cells were harvested and the mean fluorescence intensity of each sample was determined by flow cytometry. All experiments were performed in triplicates.

To further confirm the important role of the CRGDfK peptide peptides in promoting tumor cell uptake, cells (4 T1 or NIH3T3) were seeded into 12-well culture plates at 2 × 10^5^ cells per well, cultured overnight, free CRGDfK peptide peptides were added at 50 μg/mL for 1 h at 4 °C, and then incubated with RPAE-QMD at 1 μg/mL of DiD at pH 7.4 at 37 °C. After 4 h, the cells were harvested and the mean fluorescence intensity of each sample was determined by flow cytometry. All experiments were performed in triplicates.

### 2.5 Tumor penetration of RPAE-QM in vitro

In vitro tumor penetration of RPAE-QM was determined using transwell-mediated assays. The top chamber of inserts (24-well, pore size, 8 μm, Costar) was coated with 60 μL of Matrigel (Beyotime, China) diluted in serum-free medium to simulate the extracellular matrix of the tumor microenvironment in vitro. Activated NIH3T3 as the stromal cell was seeded into the upper chambers. The metastatic 4 T1 breast cancer cells were cultivated in the lower chambers. After 24 h of incubation, the nanosystem (RPAE-QMD (pH 7.4, pH 6.5), PAE-QMD, RPAE-QD and RPAE-MD) was added to the upper chambers of the inserts at 1 μg/mL DiD for another 4 h incubation. Then, 4 T1 cells were harvested, and the mean fluorescence intensity in each sample was determined by flow cytometry. All experiments were performed in triplicates. Drug-free DiD-loaded nanosystems (RPAE-D in pH 7.4 or 6.5, PAE-D) were used to confirm the reliability of the Transwell chamber model and the tumor penetration ability of blank carriers.

### 2.6 Quantitative analysis of collagen and fibronectin secretion

CAFs were activated and seeded at a density of 1 × 10^5^ cells per well in a 24-well plate. Following overnight incubation, the original culture medium was replaced with serum-free medium. The experimental groups were established as follows: Saline, RPAE-Q, RPAE-M, PAE-QM, RPAE-QM, and RPAE-QM (pH 6.5). In the serum-free medium, the concentration of DM1 was set at 10 ng/mL, and the concentration of Que. was set at 100 ng/mL. The saline group served as the control. After an additional 24 h of incubation, the supernatant was collected. Following centrifugation to remove debris, the hydroxyproline (HYP) content in the supernatant was measured using a HYP Content Assay Kit (BC0255, Solarbio). Simultaneously, the fibronectin content in the supernatant was assessed using a Mouse Fibronectin ELISA Kit (ZN2615, Beijing Biaoleibo Technology Co., Ltd.) to evaluate the fibronectin secretion levels in each group following drug treatment.

### 2.7 Inhibitory effects on cell migration and invasion activities

The inhibitory effect of RPAE-QM on the migration and invasion ability of cancer cells induced by CAFs was determined through transwell assay. Firstly, 2 × 10^5^ NIH3T3 cells in 600 μL culture medium containing 10% FBS (CMF) was added to the lower chambers. After overnight cultivation and activation, nanosystems (Saline, RPAE-Q, RPAE-M, RPAE-QM (pH 7.4, 6.5), and PAE-QM) were added at 0.1 μg/mL or equivalent concentrations of DM1 for another 4 h. Then, the drug containing supernatant was replaced with fresh culture medium. Next, for the cell migration assay, 4 T1 cells in 200 μL CMF were seeded into the top chamber of inserts (24-well, pore size, 8 μm, Costar) at 2 × 10^5^ cells per well. For the cell invasion assay, 60 μL of Matrigel (Beyotime, China) diluted in serum-free medium was added to the inserts and incubated for 30 min before 200 μL CMF with 2 × 10^5^ 4 T1 cells was added. Saline-treated cells were used as negative controls. After 24 h, the cells that migrated or invaded across the membrane were stained with crystal violet and imaged under a microscope (IX73, Olympus, Japan). The crystal violet signals for each treatment were analyzed using the ImageJ software (National Institutes of Health, Bethesda, USA).

### 2.8 Cytotoxicity

The cytotoxicity of RPAE-QM was measured in 4 T1 cancer cells. Briefly, cells were seeded into 96 well plates at 3 × 10^3^ cells per well and cultured overnight. DM1, RPAE-Q, RPAE-M, RPAE-QM (pH 7.4 and pH 6.5), and PAE-QM were added to each well at serial concentrations of DM1 (0.008, 0.04, 0.2, 1, 5, and 10 μg/mL) or equivalent concentrations. After 48 h incubation, cell viability in each group was detected using thiazolyl blue tetrazolium bromide (MTT, Sigma) assays. All samples were analyzed in triplicate. The half-maximal inhibitory concentrations (*IC*_50_) were calculated by the GraphPad Prism 10.

To quantitatively evaluate the interaction between Que. and DM1, the Chou-Talalay method was employed to calculate the Combination Index (CI). 4 T1 cells were treated withRPAE-Q, RPAE-M, and RPAE-QM. The resulting dose-effect data were analyzed using CompuSyn software to generate CI values as a function of the fraction of cells affected (Fa). A CI value <1, = 1, or > 1 indicates synergism, an additive effect, or antagonism, respectively.

### 2.9 Apoptosis

4 T1 cells were seeded into 12-well culture plates at 2 × 10^5^ cells per well, cultured overnight, and then incubated with DM1, RPAE-Q, RPAE-M, RPAE-QM (pH 7.4 and pH 6.5), and PAE-QM at 10 ng/mL or equivalent concentrations of DM1. 4 T1 cells treated with saline were used as negative controls. After 24 h, the cells were harvested and stained with Annexin V-FITC/PI Cell Apoptosis Detection Kit (Beyotime, China) for flow cytometer analysis. All experiments were performed in triplicates.

### 2.10 In vitro destruction of 3D-tumor spheroids

Three-dimensional (3D) tumor spheroids were used to further evaluate the growth inhibition of the nanosystem in vitro. Briefly, 100 μL suspensions of NIH3T3 and 4 T1 cells (number ratio: 1:1) were cultured in a 1% (wt/vol) agarose gel-coated 96-well plate at 1 × 10^3^ cells per well. After 3 days, DM1, RPAE-Q, RPAE-M, RPAE-QM (pH 7.4, 6.5), and PAE-QM were added to the plates at 0.1 μg/mL of DM1 or equivalent concentration. Untreated tumor spheroids were used as negative controls. The day of administration was considered day 0, and spheroid growth was monitored for 5 days using a microscope (IX73, Olympus, Japan).

### 2.11 Ligand preparation

Quercetin (PubChem CID: 5280343) was retrieved from PubChem. Initial 3D structures were energy-minimized using ChemDraw 20.0 to achieve optimal conformation for docking.

### 2.12 Protein model preparation

Target proteins identified through transcriptomic and network analyses were modeled using AlphaFold-predicted structures. Structures were preprocessed in PyMOL (version 2.4): (1) Removal of crystallographic water molecules; (2) Addition of polar hydrogen atoms; (3) Assignment of Kollman united-atom charges.

Based on the PubMed, KEGG, and Pathway Commons databases, proteins highly expressed in CAFs of breast cancer were collected. Based on the PubMed, KEGG, and Pathway Commons databases. Subsequently, three-dimensional structural models of these proteins were constructed using AlphaFold-predicted structures, ultimately establishing a protein target library associated with breast cancer CAFs.

### 2.13 Molecular docking using Smina

Based on the defined grid space, random sampling was performed to generate diverse conformations of the ligand Que., which were docked spatially against the overall structures of all proteins in the target protein library. During the initial docking phase, the modified Vina scoring function integrated in Smina (Version 2020.12.10) was employed to evaluate ligand-receptor binding affinity by calculating intermolecular interaction energies. Following initial ligand placement, local conformational optimization was conducted. The protein-ligand complex with optimal affinity was ultimately selected through binding energy recalculation. For Smina docking (derived from AutoDock Vina), the exhaustiveness parameter was set to 256, protein-specific parameters are listed in Table S1 (Supporting Information), and all other parameters retained default settings.

### 2.14 Molecular dynamics simulations to confirm Que. interactions with potential target protein

Molecular dynamics simulations (MDS) were performed using GROMACS 2025.1 (CUDA 12.8) with the TIP3P water model. The protein and ligand were parameterized with the CHARMM36m force field and CHARMM General Force Field, respectively. The system was solvated in a cubic box, maintained at 310.15 K and 1.0 bar, and simulated for 100 ns. These parameters were incorporated into the system topology files. Trajectory analysis was conducted using built-in software tools, including calculations of root-mean-square deviation (RMSD), root-mean-square fluctuation (RMSF), radius of gyration (Rg), hydrogen bonds, Δ molecular mechanics poisson-Boltzmann surface area (ΔMMPBSA) and free energy landscapes.

### 2.15 Assessment of MMP-3 and TGF-*β*_1_ secretion

Activated CAFs were seeded at a density of 1 × 10^5^ cells per well in a 24-well plate. After an overnight incubation, the original culture medium was replaced with serum-free medium. The cells were subsequently treated with RPAE-Q containing various concentrations of Que. (0, 0.1, 1, and 10 μg/mL) for 24 h. Following treatment, the supernatant was collected and centrifuged to remove cellular debris. The secretion of MMP-3 by CAFs was assessed using a Mouse Matrix Metalloproteinase 3 ELISA Kit (ZN2706, Beijing Biaoleibo Technology Co., Ltd.). Furthermore, the secretion of TGF-*β*_1_ by CAFs was measured using a Mouse Transforming Growth Factor-*β*_1_ ELISA Kit (ZN2762, Beijing Biaoleibo Technology Co., Ltd.) to evaluate the effects of different Que. concentrations on the secretion of these proteins.

### 2.16 Evaluating the expression of phospho-Smad2/3 proteins

Activated CAFs were seeded at a density of 1 × 10^5^ cells per well in a 24-well plate. After overnight incubation, the original culture medium was replaced with serum-free medium. The cells were treated with RPAE-Q containing different concentrations of Que. (0, 0.1, 1, and 10 μg/mL) for 24 h. The cells were harvested, washed with PBS, fixed with 4% paraformaldehyde and permeabilized with BeyoFC™ Cell Permeabilization and Wash Buffer for Flow Cytometry (Triton X-100), incubated with anti-rabbit Phospho-Smad2 (Ser465/467) + Smad3 (Ser423/425) primary antibodies (AF5920, Beyotime) at 1/200 dilution. A Cy3-labeled Goat Anti-Rabbit IgG (H + L) (A0516, Beyotime) at 1/500 dilution was used as the secondary antibody. Cells treated with saline were used as negative controls. The mean fluorescence intensity of each sample was determined by flow cytometry. All experiments were performed in triplicates.

### 2.17 Statistical analysis

All quantitative data are presented as the mean ± standard deviation (SD) from at least three independent experiments (*n* = 3), unless otherwise specified. Statistical comparisons between two groups were performed using a two-tailed, unpaired Student's *t*-test. For comparisons involving more than two groups, a one-way analysis of variance (ANOVA) was performed, followed by Tukey's post-hoc test for multiple pairwise comparisons. Differences were considered statistically significant when the *p*-value was less than 0.05. Significance levels are indicated in the figures as * for *P* < 0.05, ** for *P* < 0.01, *** for *P* < 0.001 and **** for *P* < 0.0001. All analyses were performed using GraphPad Prism software (Version 10.2.0, GraphPad Software, USA).

## 3 Results and discussion

### 3.1 Characterization of RPAE-QM

**Scheme 1:**
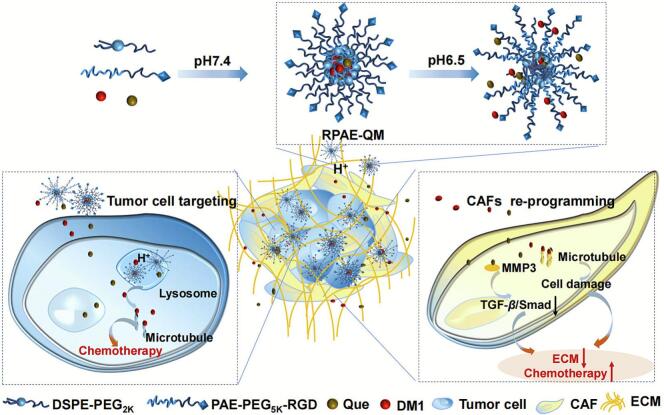
Schematic illustration of pH-responsive RPAE-QM in acidic tumor microenvironment and the corresponding mechanism of enhancing breast cancer chemotherapy.

The RPAE-QM nanoparticle, shown in Scheme 1, is self-assembled from DSPE-PEG2k and a custom PAE-PEG5k-RGD polymer, and is co-loaded with the chemotherapeutic DM1 and the potential stromal-modulating agent Que. The RGD peptide on its surface is intended to provide active tumor targeting, while the PAE backbone is designed to facilitate payload release in the acidic TME. To evaluate the function of each component, three control formulations were also prepared: PAE-QM (lacking the RGD-targeting), RPAE-Q (containing only Que), and RPAE-M (containing only DM1).

**Fig. 1:**
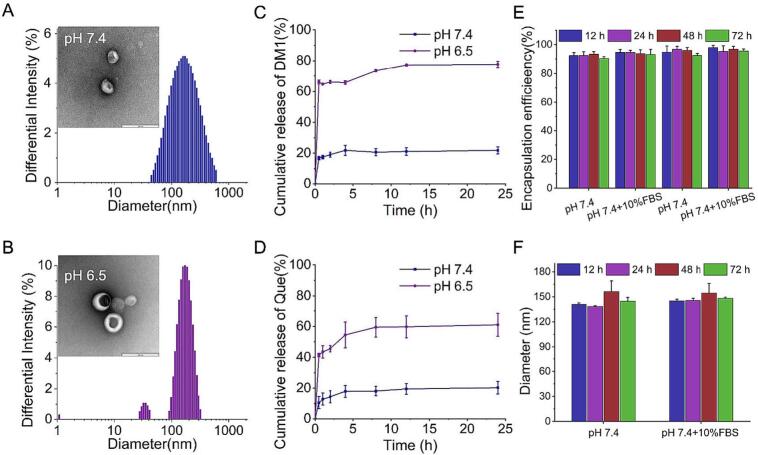
Characterization, Drug release profiles and Stability Of RPAE-QM. (A-B) Hydrodynamic size and TEM images in pH 7.4 or 6.5, scale bar = 200 nm. (C) DM1 and (D) Que. release profiles of RPAE-QM at different pH with increasing time (*n* = 3). (E) Particle size and (F) EE of RPAE-QM in PBS (pH 7.4) and PBS (pH 7.4) containing 10% FBS over time (n = 3).

The physicochemical properties and pH-responsive nature of the nanoparticles were characterized. At a physiological pH of 7.4, TEM showed that RPAE-QM formed well-defined spherical structures. However, upon exposure to an acidic pH of 6.5, which mimics the tumor microenvironment, the nanoparticles appeared structurally compromised and swollen, indicating pH-induced destabilization (Fig. 1A). This pH-induced swelling was quantified by DLS. At pH 7.4, RPAE-QM had a hydrodynamic diameter of 146.1 ± 2.57 nm and a nearly neutral surface charge (zeta potential of −0.22 ± 0.53 mV). In contrast, at pH 6.5, the average diameter significantly increased to 182.0 ± 1.0 nm (Fig. 1B, Table 1). Notably, this size increase was a consistent characteristic for all formulations containing the PAE polymer, as detailed in Table 1. This result indicates that the protonation of the PAE backbone in acidic conditions leads to polymer chain hydration and swelling, causing a structural destabilization of the nanoparticle. This engineered response is the intended mechanism to trigger the release of the encapsulated payloads, DM1 and Que., within the acidic TME.

**Table 1:** The Zeta potential, particle size and EE of the various nanoparticles (n = 3).

	Zeta potential (mV)	particle size(nm)		EE(%)	
		pH 7.4	pH 6.5	Que	DM1
RPAE-Q	0.04 ± 0.64	148.7 ± 0.75	195.7 ± 9.55	95.3 ± 4.1	/
RPAE-M	0.11 ± 0.55	64.7 ± 3.01	129.7 ± 20.9	/	96.3 ± 0.8
RPAE-QM	−0.22 ± 0.53	146.1 ± 2.57	182.0 ± 1.0	97.5 ± 4.6	94.9 ± 0.7
PAE-QM	0.95 ± 5.75	165.9 ± 1.36	208.2 ± 1.0	94.7 ± 3.7	96.0 ± 0.7

The pH-triggered swelling of RPAE-QM directly correlated with an environment-dependent drug release profile (Fig. 1C, D). The nanosystem exhibited excellent payload retention at physiological pH 7.4, resulting in minimal drug leakage during simulated circulation. Conversely, exposure to an acidic TME-mimicking environment (pH 6.5) triggered the rapid and simultaneous release of both Que. and DM1, with approximately 60% of the total payload deployed within 4 h. This pH-gated release is a critical design feature, supporting the rationale for the system's ability to concentrate its therapeutic action specifically at the tumor site while minimizing systemic exposure.

The suitability of RPAE-QM as a therapeutic delivery vehicle was further supported by its high drug loading capacity and colloidal stability. The system achieved near-quantitative encapsulation of both Que. (∼98%) and DM1 (∼95%) (Table 1). This high therapeutic payload was retained over a 72 h incubation in physiological buffer, even in the presence of 10% FBS to mimic blood components. During this period, no significant drug leakage (monitored by EE) or changes in particle size were observed (Fig. 1E, F). This indicates the platform's robustness under simulated physiological conditions, a prerequisite for potential in vivo applications.

### 3.2 In vitro cellular uptake of RPAE-QM

**Fig. 2:**
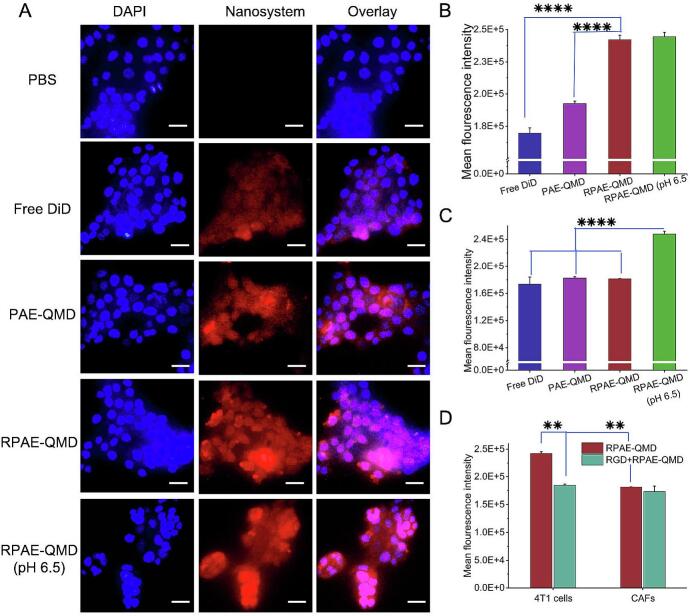
Cellular uptake of RPAE-QM in 4 T1 cells. (A) Typical images of cellular uptake of nanoparticles under the inverted fluorescence microscope, scale bar = 25 μm. (B) The quantified internalization of the various nanoparticles in 4 T1 cells, and (C) The internalization in CAFs. (D)The quantified cellular uptake of RPAE-QM in 4 T1 and CAFs in the presence and absence of the cyclic CRGDfK peptide. Data are mean ± SD (n = 3). **P < 0.01, ****P < 0.0001.

The dual-targeting capability of the system was evaluated through cellular uptake studies using DiD-labeled nanoparticles. At physiological pH 7.4, the system demonstrated RGD-dependent targeting preference: the uptake of RPAE-QMD by 4 T1 cells was significantly higher than that by CAFs, and also significantly higher than that of non-targeted PAE-QMD (Fig. 2A-2C). Notably, simulating the acidic TME (pH 6.5) resulted in a significant increase in nanoparticle internalization into the surrounding CAFs. These results are consistent with the intended dual-function design: the RGD ligand promotes preferential uptake by cancer cells, while the pH-responsive polymer enhances overall internalization into CAFs in an acidic environment.

The integrin-dependence of the observed cell-type selectivity was then investigated through a competitive blocking assay. Pre-saturating the *αvβ*_3/5_ receptors on 4 T1 cells with free cRGDfK peptide effectively negated the targeting advantage of RPAE-QMD, causing its uptake to decrease significantly (Fig. 2D). Conversely, uptake into CAFs, which have low integrin expression, remained unchanged. These observations strongly indicate that the preferential accumulation of the nanosystem in cancer cells is driven by specific RGD-integrin binding, supporting a key component of its active targeting design.

### 3.3 In vitro specific tumor penetration of RPAE-QM

**Fig. 3:**
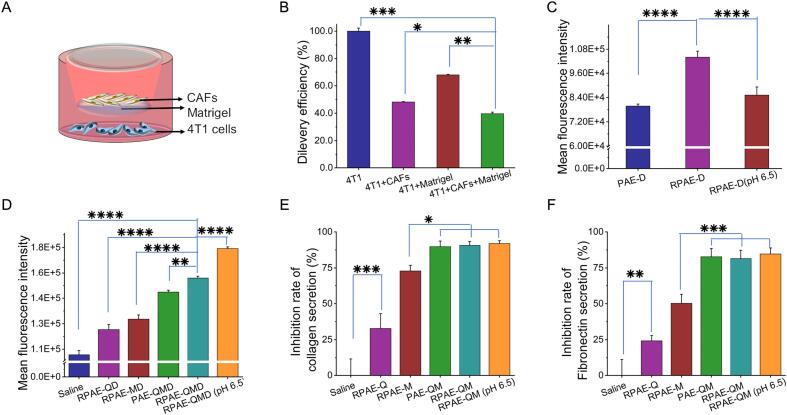
Penetration of RPAE-QM in vitro. (A) The transwell chamber model. (B-D)The mean fluorescence intensity of nanocomplex after different treatments in 4 T1 cells by flow cytometry, *n* = 3.(E)The inhibition rate of collagen secretion after different treatments in CAFs by hydroxyproline content assay kit, n = 3. (F) The inhibition rate of fibronectin secretion after different treatments in CAFs by mouse fibronectin ELISA kit, n = 3. * *P* < 0.05, **P < 0.01, ***P < 0.001 and ****P < 0.0001.

The nanoparticle's design could overcome the dense physical barriers of the TME was then evaluated. And in vitro tumor penetration model was established using a Transwell chamber, where a barrier of CAFs and Matrigel was layered above a culture of 4 T1 cancer cells (Fig. 3A). The model's suitability was first assessed by observing that the barrier effectively blocked over 60% of a simple, drug-free nanoparticle (RPAE-D) from reaching the 4 T1 cells below (Fig. 3B). This indicated the model was suitable for assessing penetrating capabilities.

Using this barrier model, the contribution of the system's design features was investigated. At physiological pH, the RGD-functionalized nanoparticle (RPAE-D) demonstrated significantly improved penetration through the barrier compared to its non-targeted counterpart (PAE-D) (Fig. 3C). This suggests that RGD-integrin binding may assist the nanoparticle in navigating past the stromal cell layer to achieve deeper delivery.

Finally, the performance of the complete, drug-loaded system was assessed. While formulations containing Que. showed some benefit at pH 7.4, the most pronounced effect was observed under acidic conditions. At a TME-mimicking pH of 6.5, RPAE-QMD achieved a level of penetration that was significantly higher than all other treatment groups (Fig. 3D). This observation suggests a synergistic effect, potentially driven by the coordinated action of the co-delivered drugs. It is hypothesized that the pH-triggered release of Que. and DM1 initiates a dual-action mechanism: Que. may act to modulate the dense CAF/ECM barrier, creating a more permissive local microenvironment that, in turn, could enhance the penetration and cytotoxic impact of the co-released DM1. This result supports the rationale behind the dual-action design within this in vitro model.

### 3.4 Inhibition of ECM production by CAFs

Following the observation of enhanced barrier penetration, the underlying mechanism was investigated by examining the system's ability to modulate ECM production. Based on the functions of Que. and DM1, it was hypothesized that their release would reduce the production of key ECM components by CAFs (Thorlacius-Ussing et al., 2024; Arpinati et al., 2024; Liang et al., 2023b). To test this, CAFs were treated with the nanoparticles, and key ECM proteins were quantified. As shown in Fig. 3E and F, the co-loaded RPAE-QM system significantly reduced the levels of both collagen (measured via hydroxyproline content) and fibronectin. This effect was greater than that of single-drug formulations, suggesting a synergistic action between Que. and DM1. Notably, the reduction was most pronounced when RPAE-QM was applied at pH 6.5, linking the effect on the matrix to the pH-triggered release of its payload. Collectively, these results indicate that RPAE-QM can suppress the production of key ECM components, providing a potential mechanistic basis for the enhanced penetration observed previously.

### 3.5 Inhibitory effects on cell migration and invasion activities enhanced by CAFs

**Fig. 4:**
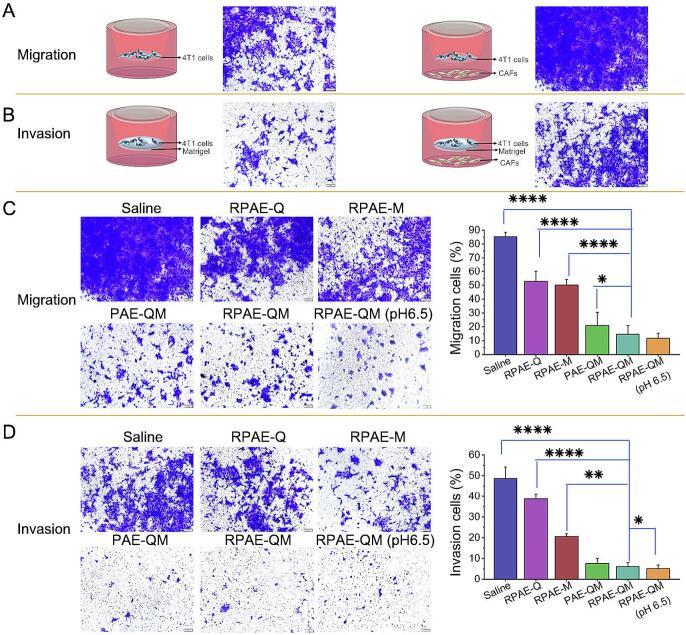
The inhibitory effect of RPAE-QM on the in vitro migration and invasion of 4 T1 cells enhanced by CAFs. (A) The typical images of migrated and (B) invaded cells across the transwell in the presence and absence of CAFs. (C)The typical images and percentage of migrated and (D) invaded cells across the transwell in the presence of the various nanoparticles treated CAFs. scale bar = 100 μm. Data are mean ± SD (n = 3). * *P* < 0.05, **P < 0.01, ***P < 0.001 and ****P < 0.0001.

Beyond physical barriers, CAFs are known to promote metastasis by secreting factors that attract cancer cells (Tiwari et al., 2022; Joshi et al., 2021). Therefore, the potential of RPAE-QM to disrupt this pro-metastatic signaling was investigated. The experimental model was first established using a Transwell assay, with CAFs in the lower chamber and 4 T1 cells in the upper chamber. As expected, the presence of untreated CAFs acted as a potent chemoattractant, significantly increasing both the migration and invasion of 4 T1 cells compared to control wells without CAFs (Fig. 4A and B). This showed the model was suitable for assessing CAF-induced migratory effects.

To assess whether the nanoparticles could disrupt the pro-tumorigenic signaling from CAFs, their effect on CAF-induced cancer cell migration and invasion was evaluated using a Transwell co-culture system. As shown in Fig. 4C and D, pre-treating the CAFs with various drug-loaded formulations significantly inhibited the subsequent migration and invasion of 4 T1 cells. Notably, a detailed comparison of the formulations revealed that this inhibitory effect was primarily driven by the stromal-modulating agent, Que. The RPAE-Q formulation demonstrated a substantially greater inhibitory effect on both migration and invasion compared to the cytotoxic agent-loaded RPAE-M. This finding directly supports the hypothesis that Que. can effectively modulate CAF behavior. Furthermore, a clear synergistic or additive effect was observed with the dual-drug RPAE-QM system, which achieved a more potent inhibition than either of the single-drug formulations. The inhibitory effect was further enhanced under acidic conditions, with RPAE-QM at pH 6.5 demonstrating the most profound reduction in both cancer cell migration. These results strongly suggest that the nanosystem, particularly through the action of Que., effectively interferes with the secretion of pro-migratory and pro-invasive factors from CAFs. By disrupting this key mechanism of stroma-cancer interaction, the RPAE-QM system validates its dual-action therapeutic strategy in this in vitro co-culture model.

### 3.6 The therapeutic effects of RPAE-QM in vitro

**Fig. 5:**
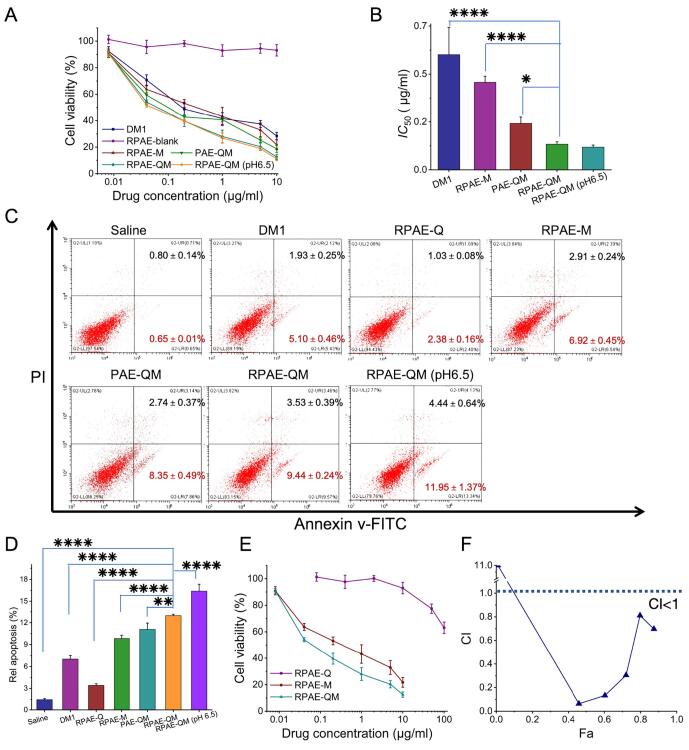
In vitro therapeutic efficacy and synergy analysis of RPAE-QM in 4 T1 cells. (A) Cell viability of 4 T1 cells incubated with different formulations for 48 h determined by MTT assay. (B) The corresponding *IC*_50_ values of the respective treatments. (C) Representative flow cytometry plots of Annexin V-FITC/PI staining for apoptosis analysis in 4 T1 cells. (D) Quantitative analysis of the total apoptotic rate. (E) Dose-response curves of free Que., free DM1, and their combination used for quantitative synergy determination. (F) The Combination Index (CI) plot as a function of Fraction affected (Fa) generated using the Chou-Talalay method; the reference line at CI = 1 indicates an additive effect, while CI < 1 indicates synergism. Data are presented as mean ± SD (n = 3). * *P* < 0.05, **P < 0.01, ***P < 0.001 and ****P < 0.0001.

The ultimate therapeutic purpose of the nanosystem—its ability to kill cancer cells—was evaluated. First, the cytotoxicity of the fully assembled nanoparticle formulations was assessed against 4 T1 cells (Fig. 5A). Analysis of the *IC*_50_ values revealed the contribution of each design feature (Fig. 5B). The results indicated that the RGD-functionalized RPAE-QM was substantially more cytotoxic than its non-targeted counterpart (PAE-QM), supporting the benefit of active targeting. Notably, the cytotoxicity of RPAE-QM was most pronounced within a TME-mimicking acidic environment. At pH 6.5, RPAE-QM achieved the lowest *IC*_50_ value of all tested formulations (0.10 ± 0.01 μg/mL). The significantly lower *IC*_50_ of the co-loaded RPAE-QM compared to the single-drug nanoparticle RPAE-M suggested a synergistic interaction between Que. and DM1.To investigate the mechanism of cell death resulting from this synergistic cytotoxicity, an apoptosis assay was performed. Consistent with the cytotoxicity data, RPAE-QM at pH 6.5 induced a significantly higher rate of apoptosis ((16.39% total apoptosis) than any other group (Fig. 5C and D).

To rigorously validate this synergistic effect, a quantitative analysis using the Chou-Talalay method was performed. Dose-response curves were generated for RPAE-Q, RPAE-M, and RPAE-QM (Fig. 5E). Based on these data, a Combination Index (CI) plot was generated (Fig. 5F). The plot clearly shows that the CI values for the Que. and DM1 combination remained consistently and significantly below 1.0 across a wide and therapeutically relevant range of effect levels (Fa values from 0.2 to 0.9), indicating a strong synergistic interaction.

It is noted that an antagonistic effect (CI > 1) was observed at a very low effect level (Fa < 0.1). This phenomenon is often attributed to the inherent limitations and higher variability of the mathematical model at the extreme lower end of the dose-response curve, where the biological effect is minimal and difficult to distinguish from experimental noise. Crucially, the data robustly demonstrate synergy throughout the therapeutically effective range, providing strong quantitative evidence that the enhanced cytotoxicity of RPAE-QM is not merely an additive effect. This validates a core principle of our therapeutic design.

Together, these results demonstrate that the system's design, which integrates targeting, quantitatively confirmed drug synergy, and environmental triggers, contributes to potent and specific cancer cell killing via apoptosis in this in vitro setting.

### 3.7 Destruction of 3D tumorsphere in vitro

**Fig. 6:**
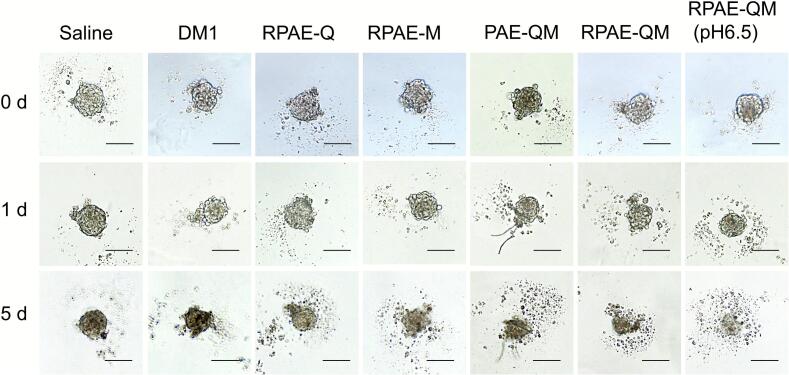
Representative images of 3D tumor spheroids after treatment with different nanoparticles, scale bar = 100 μm.

To assess the therapeutic efficacy in a model with greater physiological relevance, the ability of RPAE-QM to affect 3D tumor spheroids was evaluated. As hypothesized, the nanoparticle system demonstrated an ability to inhibit spheroid growth and disrupt their structure over a five-day period (Fig. 6). This complex model allowed for further evaluation of the system's key design features. A clear synergistic advantage was observed, as the dual-drug RPAE-QM began to affect the spheroids more effectively than monotherapies from day one. Furthermore, the system's pH-responsiveness was a key factor in this dense, multi-cellular model. At a TME-mimicking pH of 6.5, RPAE-QM induced a significantly more pronounced collapse of the tumor spheroids compared to the same treatment at pH 7.4. This suggests that the acidic environment, by triggering the release of Que. and DM1, is important for enabling the nanoparticle to penetrate deep into the 3D mass and exert its therapeutic effect. The performance of RPAE-QM in this advanced in vitro model further supports the rationale of the dual-action delivery strategy, particularly within a model system designed to mimic some of the physical challenges of solid tumors.

### 3.8 Verification of interactions between Que. and potential targets through molecular docking

To explore the potential the molecular mechanism behind Que.'s ECM-remodeling activity, a large-scale computational screen was performed, docking Que. against a library of 60 proteins. The screen revealed that Que. exhibited high-affinity binding to several key targets (full results in Table S2).

Notably, among the ten proteins with the highest binding affinity, five are central regulators of ECM homeostasis (Table 2). These high-affinity targets included Matrix Metalloproteinase-3 (MMP3; −10.5 kcal/mol), Lysyl Oxidase (LOX; −10.4 kcal/mol), LOXL2 (−10.0 kcal/mol), MMP2 (−9.9 kcal/mol), and TGF-*β*1 (−9.9 kcal/mol). The identification of this cluster of ECM-related proteins provides a potential molecular basis for Que.'s ability to reduce collagen and fibronectin secretion.

**Table 2:** Top 10 CAFs-associated proteins in breast cancer tumor microenvironment ranked by Que. docking energy and their biological functions.

Sequence number	Protein name	Protein name (Abbreviation)	Binding affinity (kcal//mol)	Functions related to CAFs in breast cancer
1	5’-Nucleotidase Ecto	CD73	−10.8	Immunosuppression (Yu et al., 2021; Bi et al., 2025)
2	Matrix Metallopeptidase 3	MMP3	−10.5	ECM remodeling (Xu et al., 2024; Liu et al., 2025a)
3	Lysyl Oxidase	LOX	−10.4	ECM remodeling (Sleeboom et al., 2024; Liu et al., 2025a)
4	Neprilysin	CD10	−10.2	CAF-subset marker (Erler et al., 2006)
5	Indoleamine 2,3- Dioxygenase 1	IDO1	−10.1	Immunosuppression (Hu et al., 2022; Tsoumakidou, 2023)
6	Lysyl Oxidase Like 2	LOXL2	−10	ECM remodeling, angiogenesis (Sleeboom et al., 2024; Liu et al., 2025b)
7	Matrix Metallopeptidase 2	MMP2	−9.9	ECM remodeling, angiogenesis (Xu et al., 2024; Liu et al., 2025b)
8	Transforming Growth Factor beta 1	TGF-*β*1	−9.9	CAF activation, ECM remodeling, immunosuppression (Kennel et al., 2023; Flynn et al., 2025)
9	Monocarboxylate Transporter 4	MCT4	−9.8	Promoting lactate efflux (Mayorca-Guiliani et al., 2025)
10	Hepatocyte Growth Factor	HGF	−9.8	Induction of epithelial- mesenchymal transition (EMT) in cancer cells (Liu et al., 2024)

**Fig. 7:**
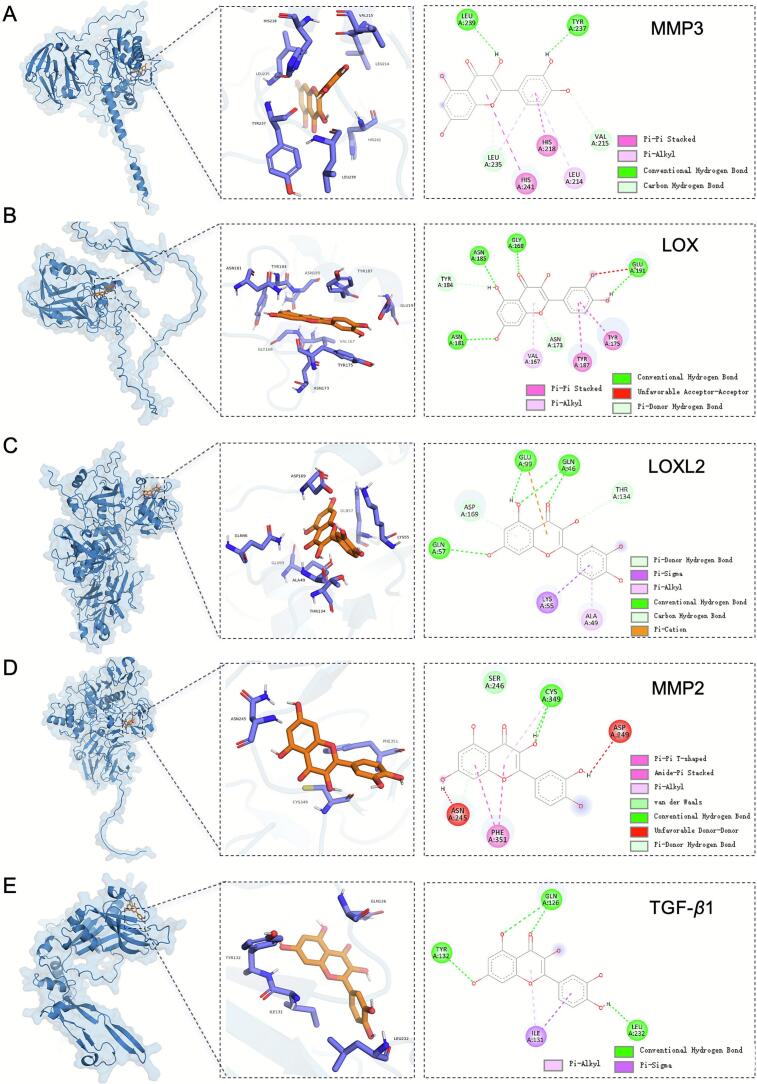
Two- and three-dimensional mapping of the binding sites between Que. and five proteins by Smina docking: (A) MMP3, (B) LOX, (C) LOXL2, (D) MMP2, and (E) TGF-*β*1. Que. is displayed in orange, while the target proteins are shown in blue-violet. (For interpretation of the references to colour in this figure legend, the reader is referred to the web version of this article.)

Further analysis of the binding poses revealed that Que.'s high affinity for these targets is stabilized by a network of non-covalent interactions. As shown for the top-ranked protein, MMP3, and the other key targets (Fig. 7A-E), the binding is primarily mediated by a combination of conventional hydrogen bonds, carbon‑hydrogen bonds, and pi-alkyl interactions. This computational analysis identifies a plausible mechanism by which Que. may interact with key enzymatic and signaling pathways responsible for ECM synthesis and maintenance, providing a strong rationale for the subsequent experimental validation.

### 3.9 Verification of binding capacity through MDS

**Fig. 8:**
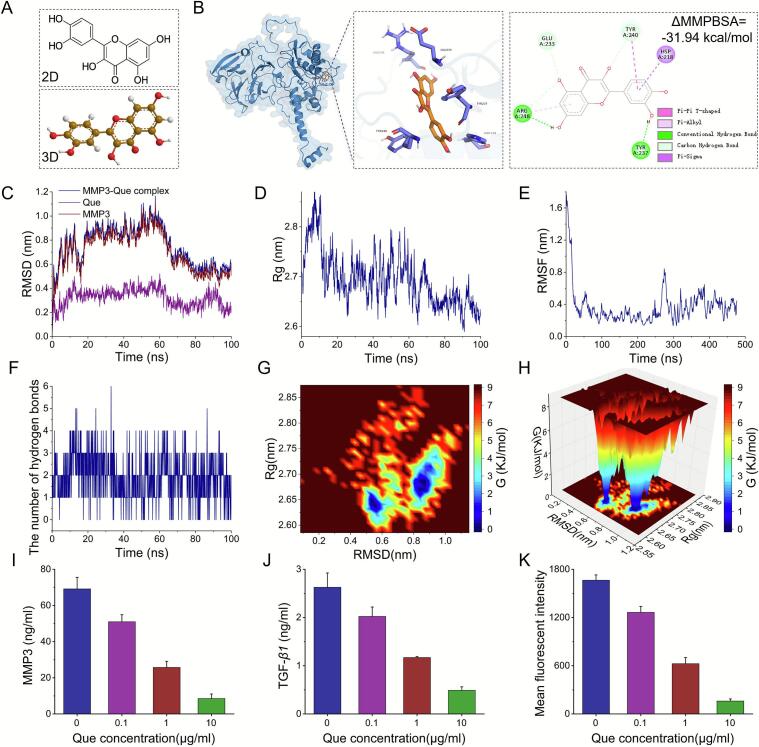
Results of molecular dynamics simulations (MDS) involving Que. and MMP3. (A) Structural formula of Que. from the PubChem database. (B) Two- and three-dimensional mapping of the binding sites, and ΔMMPBSA between Que. and MMP3 by MDS on 80 ns. (C) The RMSD values for each target MMP3-Que complex. (D) Rg rate curve of the protein-Que complex. (E) Variations in protein flexibility throughout the Que. simulation. (F) Dynamics of hydrogen bonding as observed in the molecular dynamics simulations. (G-H) Two-dimensional and three-dimensional mappings of the free energy landscape. (I-J) The MMP3 and TGF-β1 secretion after different treatments in CAFs by assay kit, n = 3. (K) The phospho-Smad2/3 proteins expression after different treatments in CAFs through flow cytometer analysis, n = 3. ΔMMPBSA: Δ molecular mechanics poisson-Boltzmann surface area; RMSD: root-mean-square deviation; Rg: radius of gyration; RMSF: root-mean-square fluctuation.

Having identified MMP3 as a top-ranked target in static docking with a highly favorable binding free energy (ΔG = −31.94 kcal/mol, Fig. 8B), we next performed a 100 ns all-atom molecular dynamics simulation to the theoretical stability of this interaction. The two-dimensional and three-dimensional structures of Que. are depicted in Fig. 8A. Our analysis first focused on the structural integrity of the complex at both global and local levels. Globally, the RMSD of the complex's backbone reached equilibrium after approximately 80 ns, maintaining exceptional stability for the remainder of the simulation (Fig. 8C). This was corroborated by a stable Rg, which confirmed that the protein maintained its compact, folded structure without unfolding (Fig. 8D). To examine local dynamics, we analyzed the RMSF for each amino acid residue (Fig. 8E). The RMSF plot reveals that while specific loop regions exhibit expected flexibility, the residues constituting the protein's core architecture and, critically, the binding pocket, maintain low fluctuation, indicating high structural rigidity. This overall stability is driven by a persistent network of non-covalent interactions, including the consistent presence of hydrogen bonds throughout the simulation (Fig. 8B and F).

To further quantify the system's thermodynamic stability, we constructed a free energy landscape. The landscape revealed a bistable conformational ensemble, characterized by two distinct and deep energy wells separated by a high energy barrier (Fig. 8G and H). This suggests the complex can adopt highly stable, long-lived, low-energy conformations. Collectively, these simulation results support our initial docking study, suggesting that Que. can form a structurally stable and enduring complex with MMP3. This provides a strong theoretical rationale for investigating MMP3 as a key component of Que.'s mechanism of action in subsequent biological experiments.

### 3.10 Molecular target validation of Que. in CAFs

Our computational modeling first allowed us to formulate a precise, testable hypothesis regarding Que.'s mechanism of action. Molecular docking identified a cluster of five ECM-regulating proteins as top-binding targets, all of which are linked to the TGF-*β* signaling pathway (Jia et al., 2025; Krstic and Santibanez, 2014). Subsequent MDS analysis confirmed that the highest-affinity target, MMP3, forms an exceptionally stable complex with Que. Given that MMPs are key upstream activators of TGF-*β* signaling, we hypothesized that Que. remodels the stroma by suppressing the MMP3-mediated TGF-*β*/Smad2/3 signaling axis.

To experimentally validate this computational hypothesis, we treated CAFs with increasing concentrations of Que. and measured the protein expression of the key pathway components. Consistent with our hypothesis, ELISA and flow cytometry analysis revealed a clear, dose-dependent decrease in the secretion/expression of MMP3, TGF-*β*1, and Smad2/3 (Figs. 8I-K). The simultaneous downregulation of these three critical proteins provides strong experimental support for the idea that Que. effectively inhibits this signaling cascade. These findings, which demonstrate a reduction in MMP3 expression and downstream signaling, are consistent with our computational model and support the conclusion that Que.'s mechanism involves the modulation of the MMP3/TGF-*β*/Smad axis. By identifying the suppression of this signaling pathway as a key mechanism, we provide a clear rationale for Que.'s ability to remodel the tumor microenvironment, validating its strategic inclusion in our therapeutic nanosystem.

### 3.11 Study limitations and future perspectives

While this study provides a strong proof-of-concept for the dual-action nanosystem, several limitations should be acknowledged, as they highlight important directions for future research. A key limitation is the use of TGF-*β*-induced NIH3T3 cells as a CAF model. This model, while suitable for initial validation, does not fully recapitulate the complex heterogeneity of clinical CAF populations. Future studies employing more physiologically relevant models, such as patient-derived primary CAFs, are essential to confirm these findings and enhance their translational relevance.

Furthermore, the scope of this work is entirely confined to in vitro investigations. The absence of in vivo data means that the nanoparticle's biodistribution, safety profile, and therapeutic efficacy in a complex biological system remain to be determined. Therefore, a crucial next step will be to evaluate the RPAE-QM nanosystem in appropriate animal models of fibrotic tumors.

Finally, the statistical power of the study warrants consideration. The sample size for most experiments was limited to *n* = 3, a constraint of the experimental setup, which increases the risk of Type II errors. It is important to note, however, that even with this limitation, analysis using ANOVA for multi-group comparisons consistently identified clear and statistically significant differences for the primary endpoints. The conclusions drawn from these data therefore represent a compelling, albeit preliminary, foundation. Confirmation of these findings with greater statistical confidence will require follow-up studies employing larger sample sizes.

## 4 Conclusion

In this study, a pH-responsive, dual-drug nanosystem, RPAE-QM, was designed and evaluated for its potential to address the barriers of the TME. By co-delivering the chemotherapeutic DM1 and the potential stromal-remodeling agent Que., RPAE-QM exhibited synergistic cytotoxicity in both 2D cell cultures and 3D tumor spheroid models. Notably, its therapeutic effect was significantly enhanced under acidic conditions mimicking the TME, suggesting that pH-triggered drug release is an important factor for affecting dense multicellular structures in vitro. To investigate the mechanism underpinning this stromal modulation, computational modeling was combined with experimental assays. Our findings suggest that Que.'s therapeutic effect may be mediated, at least in part, by the suppression of the pro-fibrotic MMP3/TGF-*β*/Smad2/3 signaling pathway in CAFs, thereby interfering with the machinery responsible for excessive ECM production. By integrating a targeted cytotoxic agent with a stromal-modulating agent into a responsive delivery vehicle, this work presents a promising strategy to simultaneously target physical and cellular barriers within the TME in vitro. These findings provide a rationale for the coordinated, dual-action therapeutic concept of RPAE-QM. While acknowledging the limitations of the current in vitro models and sample sizes, this study provides a mechanistic foundation and a clear rationale for the future in vivo evaluation of RPAE-QM. Such studies will be critical to determine if this pre-clinical strategy can translate into a safe and effective therapeutic approach for fibrotic solid tumors.

Chemical Compounds and Key Biological Molecules Studied.

1. Quercetin
2. Mertansine (DM1)
3. Poly(*β*-amino ester) (PAE)
4. DSPE-PEG2000-RGD
5. Transforming growth factor beta 1 (TGF-*β*_1_)
6. Matrix metalloproteinase-3 (MMP-3)
7. Collagen
8. Fibronectin
9. Phospho-Smad2/3
10. Thiazolyl Blue Tetrazolium Bromide (MTT)

## CRediT authorship contribution statement

**Tao Tan:** Writing – review & editing, Writing – original draft, Visualization, Validation, Supervision, Methodology, Investigation, Formal analysis, Data curation, Conceptualization. **Yihan Wang:** Visualization, Validation, Methodology. **Ran Cheng:** Visualization, Validation, Methodology. **Dongsheng Yang:** Writing – review & editing, Project administration, Funding acquisition.

## Declaration of competing interest

The authors declare that they have no known competing financial interests or personal relationships that could have appeared to influence the work reported in this paper.

## Data Availability

Data will be made available on request.

## Appendix A Supplementary data

Supplementary material: Supporting Information Table S1. Parameters for molecular docking analysis of target proteins using smina. Table S2. Molecular docking results showing the binding affinities (kcal/mol) between Quercetin (Que) and various target proteins. Image 1

## References

[bb0005] Arpinati L., Carradori G., Scherz-Shouval R. (2024). CAF-induced physical constraints controlling T cell state and localization in solid tumours. Nat. Can..

[bb0010] Bi C., Patel J.S., Liang S.H. (2025). Development of CD73 inhibitors in tumor immunotherapy and opportunities in imaging and combination therapy. J. Med. Chem..

[bb0015] Cai X., Fang Z., Dou J., Yu A., Zhai G. (2013). Bioavailability of quercetin: problems and promises. Curr. Med. Chem..

[bb0025] Chhabra Y., Weeraratna A.T. (2023). Fibroblasts in cancer: Unity in heterogeneity. Cell.

[bb0030] De Visser K.E., Joyce J.A. (2023). The evolving tumor microenvironment: from cancer initiation to metastatic outgrowth. Cancer Cell.

[bb0035] Duan H., Liu C., Hou Y., Liu Y., Zhang Z., Zhao H., Xin X., Liu W., Zhang X., Chen L., Jin M., Gao Z., Huang W. (2022). Sequential delivery of quercetin and paclitaxel for the fibrotic tumor microenvironment remodeling and chemotherapy potentiation via a dual-targeting hybrid micelle-in-liposome system. ACS Appl. Mater. Interfaces.

[bib196] Erler J.T., Bennewith K.L., Nicolau M., Dornhöfer N., Kong C., Le Q.T., Chi J.T., Jeffrey S.S., Giaccia AJ. (2006). Lysyl oxidase is essential for hypoxia-induced metastasis. Nature.

[bb0040] Flynn J.M., Thadani N., Gallagher E.E., Azzaro I., Bodnar C.M., McCarty C.P., Romano G., Webster M.R., Capparelli C. (2025). Plasticity and functional heterogeneity of cancer-associated fibroblasts. Cancer Res..

[bb0045] Ge Z., Xu M., Ge Y., Huang G., Chen D., Ye X., Xiao Y., Zhu H., Yin R., Shen H., Ma G., Qi L., Wei G., Li D., Wei S., Zhu M., Ma H., Shi Z., Wang X., Ge X., Qian X. (2023). Inhibiting G6PD by quercetin promotes degradation of EGFR T790M mutation. Cell Rep..

[bb0050] Hu D., Li Z., Zheng B., Lin X., Pan Y., Gong P., Zhou W., Hu Y., Chen C., Chen L., Zhou J., Wang L. (2022). Cancer-associated fibroblasts in breast cancer: challenges and opportunities. Cancer Commun.

[bb0055] Jia H., Chen X., Zhang L., Chen M. (2025). Cancer associated fibroblasts in cancer development and therapy. J. Hematol. Oncol..

[bb0060] Joshi R.S., Kanugula S.S., Sudhir S., Pereira M.P., Jain S., Aghi M.K. (2021). The role of cancer-associated fibroblasts in tumor progression. Cancers.

[bb0065] Kennel K.B., Bozlar M., De Valk A.F., Greten F.R. (2023). Cancer-associated fibroblasts in inflammation and antitumor immunity. Clin. Cancer Res..

[bb0070] Krstic J., Santibanez J.F. (2014). Transforming growth factor-beta and matrix metalloproteinases: Functional interactions in tumor stroma-infiltrating myeloid cells. Sci. World J..

[bb0075] Li Y., Shen X., Ding H., Zhang Y., Pan D., Su L., Wu Y., Fang Z., Zhou J., Gong Q., Luo K. (2024). Dendritic nanomedicine enhances chemo-immunotherapy by disturbing metabolism of cancer-associated fibroblasts for deep penetration and activating function of immune cells. Acta Phasrm. Sin. B.

[bb0080] Liang T., Tao T., Wu K., Liu L., Xu W., Zhou D., Fang H., Ding Q., Huang G., Wu S. (2023). Cancer-associated fibroblast-induced remodeling of tumor microenvironment in recurrent bladder cancer. Adv. Sci..

[bb0085] Liang D., Liu L., Zhao Y., Luo Z., He Y., Li Y., Tang S., Tang J., Chen N. (2023). Targeting extracellular matrix through phytochemicals: a promising approach of multi-step actions on the treatment and prevention of cancer. Front. Pharmacol..

[bb0090] Lin W., Jiang X. (2025). Innate immune activation with multifunctional nanoparticles for cancer immunotherapy. Angew. Chem. Int. Ed..

[bb0095] Liu S., Zhang X., Wang W., Li X., Sun X., Zhao Y., Wang Y., Li Y., Hu F., Ren H. (2024). Metabolic reprogramming and therapeutic resistance in primary and metastatic breast cancer. Mol. Cancer.

[bb0100] Liu Y., Sinjab A., Min J., Han G., Paradiso F., Zhang Y., Wang R., Pei G., Dai Y., Liu Y., Cho K.S., Dai E., Basi A., Burks J.K., Rajapakshe K.I., Chu Y., Jiang J., Zhang D., Yan X., Guerrero P.A., Serrano A., Li M., Hwang T.H., Futreal A., Ajani J.A., Soto L.M.S., Jazaeri A.A., Kadara H., Maitra A., Wang L. (2025). Conserved spatial subtypes and cellular neighborhoods of cancer-associated fibroblasts revealed by single-cell spatial multi-omics. Cancer Cell.

[bb0105] Liu H., Sun X., Dong B., Zhang J., Zhang J., Gu Y., Chen L., Pang X., Ye J., Wang X., Rong Z. (2025). Systematic characterisation and analysis of Lysyl oxidase family members as drivers of tumour progression and multiple drug resistance. J. Cell. Mol. Med..

[bb0110] Mayorca-Guiliani A.E., Leeming D.J., Henriksen K., Mortensen J.H., Nielsen S.H., Anstee Q.M., Sanyal A.J., Karsdal M.A., Schuppan D. (2025). ECM formation and degradation during fibrosis, repair, and regeneration. npj Metab. Health Dis..

[bb0115] Meng S., Cao Y., Lu L., Li X., Sun S., Jiang F., Lu J., Fan D., Han X., Yao T. (2024). Quercetin promote the chemosensitivity in organoids derived from patients with breast cancer. Breast Cancer Targets Ther..

[bb0120] Nguyen L.N., Ngo W., Lin Z.P., Sindhwani S., MacMillan P., Mladjenovic S.M., Chan W.C. (2024). The mechanisms of nanoparticle delivery to solid tumours. Nat. Rev. Bioeng..

[bb0125] Ouyang C., Zhang W., Nie J., Yu L., Liu J., Ren L., Chen G. (2023). Nanoparticles with active targeting ability and acid responsiveness for an enhanced antitumor effect of docetaxel. Biomacro.

[bb0130] Sleeboom J.J., van Tienderen G.S., Schenke-Layland K., van der Laan L.J., Khalil A.A., Verstegen M.M. (2024). The extracellular matrix as hallmark of cancer and metastasis: from biomechanics to therapeutic targets. Sci. Transl. Med..

[bb0135] Sun L., Liu H., Ye Y., Lei Y., Islam R., Tan S., Tong R., Miao Y., Cai L. (2023). Smart nanoparticles for cancer therapy. Signal Trans. Target. Ther..

[bb0140] Tan T., Hu H., Wang H., Li J., Wang Z., Wang J., Wang S., Zhang Z., Li Y. (2019). Bioinspired lipoproteins-mediated photothermia remodels tumor stroma to improve cancer cell accessibility of second nanoparticles. Nat. Commun..

[bb0145] Tan T., Chang W., Wang T.L., Chen W., Chen X., Yang C., Yang D. (2024). pH-responsive charge-reversal smart nanoparticles for Co-delivery of mitoxantrone and copper ions to enhance breast cancer chemo-chemodynamic combination therapy. Int. J. Nanomedicine.

[bb0150] Thorlacius-Ussing J., Jensen C., Nissen N.I., Cox T.R., Kalluri R., Karsdal M., Willumsen N. (2024). The collagen landscape in cancer: profiling collagens in tumors and in circulation reveals novel markers of cancer-associated fibroblast subtypes. J. Pathol..

[bb0155] Tiwari A., Trivedi R., Lin S.Y. (2022). Tumor microenvironment: barrier or opportunity towards effective cancer therapy. J. Biomed. Sci..

[bb0160] Tsoumakidou M. (2023). The advent of immune stimulating CAFs in cancer. Nat. Rev. Cancer.

[bb0165] Wang X., Zhang H., Chen X., Wu C., Ding K., Sun G., Luo Y., Xiang D. (2023). Overcoming tumor microenvironment obstacles: current approaches for boosting nanodrug delivery. Acta Biomater..

[bb0170] Wang B., Hu S., Teng Y., Chen J., Wang H., Xu Y., Wang K., Xu J., Cheng Y., Xiang G. (2024). Current advance of nanotechnology in diagnosis and treatment for malignant tumors. Signal Trans. Target. Ther..

[bb0175] Xu Y., Liu D., Zhang W., Liu Z., Liu J., Zhang W., Song R., Li J., Yang F., Wang Y., Liu D., Qian G., Tang H., Chen X., Lai Y. (2024). Discovery of novel 5-(pyridazin-3-yl) pyrimidine-2, 4 (1*H*,3*H*)-dione derivatives as potent and orally bioavailable inhibitors targeting ecto-5′-nucleotidase. J. Med. Chem..

[bb0180] Xu H., Yao Q., Hu X., Zheng D., Ren C., Ren Z., Gao Y. (2025). On-membrane supramolecular assemblies serving as bioorthogonal gating for melphalan. Angew. Chem. Int. Ed..

[bb0185] Yang P., Xu Y., Zhi X., Li R., Wang B., Liu R., Dai Z., Qian L. (2024). Photodynamically tumor vessel destruction amplified tumor targeting of nanoparticles for efficient chemotherapy. ACS Nano.

[bb0190] Yu Y., Wu X.A., Liu S., Zhao H., Li B., Zhao H., Feng X. (2021). Piezo1 regulates migration and invasion of breast cancer cells via modulating cell mechanobiological properties. Acta Biochim. Biophys. Sin..

[bb0195] Zhao P., Wang S., Jiang J., Gao Y., Wang Y., Zhao Y., Zhang J., Zhang M., Huang Y. (2023). Targeting lactate metabolism and immune interaction in breast tumor via protease-triggered delivery. J. Control. Release.

