# Supplementary material for: A pH-responsive dual-drug nanoplatform for stromal remodeling and enhanced chemotherapy via MMP3/TGF-β inhibition
Source: Int J Pharm X. 2026 Jan 13;11:100489. doi: 10.1016/j.ijpx.2026.100489 (PMC12830281; doi:10.1016/j.ijpx.2026.100489)
Supplement: Supplementary file 1 — Supplementary material: Supporting Information Table S1. Parameters for molecular docking analysis of target proteins using smina. Table S2. Molecular docking results showing the binding affinities (kcal/mol) between Quercetin (Que) and various target proteins. [file mmc1.docx]

ORIGINAL RESEARCH

**Supporting information**

**A pH-Responsive Dual-Drug Nanoplatform for Stromal Remodeling and Enhanced Chemotherapy via MMP3/TGF-*β* Inhibition**

**Tao Tan^1^, Yihan Wang^2^, Ran Cheng^2^, Dongsheng Yang^1^**

^1^College of Life Science, Zhuhai College of Science and Technology, Zhuhai 519041, People’s Republic of China;

^2^College of Life Science, Jilin University, Changchun 130012, People’s Republic of China

Correspondence: Dongsheng Yang, College of Life Science, Zhuhai College of Science and Technology, Zhuhai 519041, People’s Republic of China, Email yds@zcst.edu.cn

**Table S1** Protein parameter in the smina docking shown as below

| Protein | Center_x | Center_y | Center_z | Size_x | Size_y | Size_z |
| --- | --- | --- | --- | --- | --- | --- |
| PDGFR*β* | -8.1035 | -17.173 | -11.046 | 152.533 | 130.178 | 118.652 |
| SNAI1 | 24.0575 | -14.606 | -19.4495 | 92.837 | 93.776 | 90.141 |
| FSP1 / S100A4 | 18.194 | -11.4875 | 193.758 | 84.894 | 87.181 | 94.302 |
| PDGFRα | 17.8975 | 126.302 | 3.8555 | 106.513 | 108.818 | 96.481 |
| CAV1 | 195.6925 | 195.8675 | 205.908 | 186.841 | 187.355 | 90.058 |
| WNT7A | 7.492 | 9.4355 | -1.973 | 143.686 | 120.247 | 131.368 |
| MCT4 | -14.948 | -12.6295 | 23.7305 | 135.196 | 126.937 | 145.491 |
| CXCL14 | -18.654 | 3.5285 | 6.2515 | 127.99 | 83.911 | 111.417 |
| TIMP1 | -18.198 | 0.668 | -4.0705 | 133.922 | 90.302 | 110.227 |
| PDGF | 15.1615 | 0.217 | -5.6075 | 150.129 | 141.328 | 127.959 |
| IGF-2 | -16.6745 | -4.098 | 10.5985 | 149.075 | 99.284 | 173.137 |
| COL1A1 | -10.856 | 1.5215 | -1.207 | 209.318 | 225.667 | 212.862 |
| MMP1 | -8.7385 | -7.668 | -18.1345 | 126.939 | 118.576 | 160.805 |
| TP53 | 10.456 | 5.9105 | -16.8935 | 134.258 | 157.289 | 139.993 |
| IL-6 | -23.429 | -20.124 | 5.99 | 132.368 | 132.6 | 113.366 |
| COL1A2 | 4.7055 | -2.4465 | -5.278 | 224.741 | 206.741 | 198.436 |
| MMP2 | -17.273 | 12.1405 | 8.8155 | 147.512 | 139.655 | 147.319 |
| MMP3 | -1.2715 | 11.131 | -9.4865 | 140.593 | 135.884 | 132.655 |
| CD10 | -3.763 | 2.796 | -32.8935 | 127.862 | 113.62 | 183.949 |
| Vimentin | 23.167 | -5.133 | -8.5025 | 290.638 | 118.428 | 216.093 |
| FGF2 | -7.16 | -11.0355 | 12.125 | 162.56 | 148.695 | 147.414 |
| SPARC | -16.291 | 18.383 | -0.5975 | 132.592 | 130.308 | 151.783 |
| IL-8 / CXCL8 | 18.8135 | -10.1605 | 3.0225 | 125.771 | 113.685 | 89.795 |
| FGF5 | -8.237 | -0.2325 | 16.831 | 161.026 | 137.913 | 132.63 |
| COL6A1 | 11.5385 | -3.463 | 19.6285 | 171.531 | 176.832 | 207.291 |
| COL6A2 | 3.6845 | -21.851 | 4.158 | 179.239 | 205.648 | 204.37 |
| CCL2 / MCP-1 | -7.417 | 17.121 | -19.0825 | 93.288 | 110.176 | 126.017 |
| HGF | -5.813 | 13.425 | 0.396 | 160.112 | 150.188 | 159.562 |
| MMP9 | -4.8845 | 8.876 | -22.281 | 134.633 | 147.834 | 195.11 |
| IDO1 | -6.9845 | 1.7825 | -2.154 | 120.293 | 105.799 | 112.226 |
| TIMP2 | -3.3135 | -20.037 | -2.544 | 111.113 | 133.358 | 101.91 |
| CD73 | -15.479 | -14.0025 | 14.748 | 157.098 | 134.133 | 163.53 |
| MMP11 | 3.8465 | 9.638 | -8.994 | 168.487 | 139.42 | 147.452 |
| COL5A3 | 2.5215 | -3.21 | 2.725 | 205.117 | 221.988 | 231.794 |
| FSP1 / S100A4 | -2.2425 | -8.7765 | 0.111 | 89.029 | 99.635 | 98.56 |
| LOX | 13.4975 | -20.0575 | -14.6685 | 124.229 | 197.115 | 137.059 |
| CTGF / CCN2 | -11.44 | 2.855 | 18.031 | 133.166 | 112.76 | 181.08 |
| THBS2 | -10.2995 | 9.739 | 7.7745 | 208.937 | 158.02 | 181.245 |
| TIMP3 | -1.6855 | -22.277 | -0.8675 | 108.049 | 156.936 | 98.401 |
| STAT3 | -8.076 | 4.574 | -0.474 | 158.408 | 144.304 | 178.738 |
| WNT5A | -15.637 | 1.483 | -16.1365 | 138.886 | 109.074 | 160.853 |
| TP53 | 0.876 | -4.882 | 13.4515 | 239.578 | 153.928 | 160.275 |
| CXCL12 / SDF-1 | 10.7175 | 0.302 | -15.117 | 98.161 | 89.238 | 125.504 |
| MMP14 | -3.8705 | -17.567 | -16.444 | 134.753 | 200.03 | 164.218 |
| FXYD1 | -13.351 | 17.4215 | 0.597 | 125.786 | 106.803 | 143.294 |
| CXCR4 | -0.676 | 16.122 | 8.1295 | 135.658 | 126.952 | 167.577 |
| *α*-SMA | 8.371 | 0.9045 | 2.052 | 120.396 | 104.275 | 115.682 |
| SMAD3 | -16.3485 | 2.576 | 6.2005 | 137.915 | 113.728 | 147.891 |
| HSF1 | -27.041 | 9.938 | 18.0525 | 164.5 | 170.246 | 220.263 |
| FAP | -3.1935 | 6.3965 | 29.73 | 130.257 | 119.787 | 186.628 |
| SMAD4 | 4.1075 | 8.1655 | 2.49 | 156.249 | 183.715 | 134.472 |
| POSTN | -11.8025 | 8.37 | 8.6855 | 160.297 | 183.784 | 147.531 |
| SMAD2 | -14.7605 | 4.8265 | -10.842 | 136.391 | 147.469 | 153.25 |
| SFRP4 | -16.0685 | 14.9355 | 6.4335 | 148.439 | 149.103 | 136.491 |
| PDPN | 4.251 | -10.7795 | -10.1 | 141.504 | 132.017 | 132.492 |
| PD-L1 | 28.8885 | -15.249 | -21.136 | 182.797 | 123.91 | 207.234 |
| GPR77 | 10.165 | 0.158 | 0.099 | 153.052 | 92.532 | 141.79 |
| LOXL2 | 0.8425 | -10.2365 | 20.117 | 129.017 | 141.157 | 181.116 |
| VEGF | 12.935 | -15.312 | 2.233 | 191.04 | 188.204 | 136.982 |
| TGF-*β*1 | 21.383 | -6.742 | 12.9175 | 186.196 | 132.466 | 125.885 |

| **Table S2** The docking binding energy between Que and protein | | | |
| --- | --- | --- | --- |
| Sequence number | Protein name  (Abbreviation) | Protein name | Bindingaffinity  （kcal/mol） |
| 1 | CD73 | 5'-Nucleotidase Ecto | -10.8 |
| 2 | MMP3 | Matrix Metallopeptidase 3 | -10.5 |
| 3 | LOX | Lysyl Oxidase | -10.4 |
| 4 | CD10 | Neprilysin | -10.2 |
| 5 | IDO1 | Indoleamine 2,3-Dioxygenase 1 | -10.1 |
| 6 | LOXL2 | Lysyl Oxidase Like 2 | -10 |
| 7 | MMP2 | Matrix Metallopeptidase 2 | -9.9 |
| 8 | TGF-β1 | Transforming Growth Factor beta 1 | -9.9 |
| 9 | MCT4 | Monocarboxylate Transporter 4 | -9.8 |
| 10 | HGF | Hepatocyte Growth Factor | -9.8 |
| 11 | α-SMA | Alpha-Smooth Muscle Actin | -9.7 |
| 12 | THBS2 | Thrombospondin 2 | -9.6 |
| 13 | GPR77 | G Protein-Coupled Receptor 77 | -9.4 |
| 14 | COL5A3 | Collagen Type V Alpha 3 Chain | -9.3 |
| 15 | MMP14 | Matrix Metallopeptidase 14 | -9.3 |
| 16 | POSTN | Periostin | -9.2 |
| 17 | SMAD2 | SMAD Family Member 2 | -9.2 |
| 18 | PD-L1 | Programmed Death-Ligand 1 | -9.1 |
| 19 | WNT5A | Wnt Family Member 5A | -9.1 |
| 20 | MMP11 | Matrix Metallopeptidase 11 | -9.1 |
| 21 | COL1A2 | Collagen Type I Alpha 2 Chain | -9.1 |
| 22 | STAT3 | Signal Transducer and Activator of Transcription 3 | -9 |
| 23 | FAP | Fibroblast Activation Protein, alpha | -9 |
| 24 | TIMP1 | TIMP Metallopeptidase Inhibitor 1 | -9 |
| 25 | STAT3 | Signal Transducer and Activator of Transcription 3 | -9 |
| 26 | WNT7A | Wnt Family Member 7A | -9 |
| 27 | PDGFRα | Platelet-Derived Growth Factor Receptor alpha | -8.9 |
| 28 | MMP9 | Matrix Metallopeptidase 9 | -8.9 |
| 29 | COL6A1 | Collagen Type VI Alpha 1 Chain | -8.8 |
| 30 | TP53 | Tumor Protein P53 | -8.8 |
| 31 | VEGF | Vascular Endothelial Growth Factor A | -8.8 |
| 32 | SULF1 | Sulfatase 1 | -8.8 |
| 33 | MMP1 | Matrix Metallopeptidase 1 | -8.8 |
| 34 | SMAD4 | SMAD Family Member 4 | -8.7 |
| 35 | TIMP2 | TIMP Metallopeptidase Inhibitor 2 | -8.7 |
| 36 | SMAD3 | SMAD Family Member 3 | -8.6 |
| 37 | CXCL14 | C-X-C Motif Chemokine Ligand 14 | -8.5 |
| 38 | IL-6 | Interleukin-6 | -8.5 |
| 39 | SPARC | Secreted Protein Acidic and Cysteine Rich | -8.4 |
| 40 | TIMP3 | TIMP Metallopeptidase Inhibitor 3 | -8.4 |
| 41 | SNAI1 | Snail Family Transcriptional Repressor 1 | -8.3 |
| 42 | CTGF / CCN2 | Connective Tissue Growth Factor | -8.3 |
| 43 | SFRP4 | Secreted Frizzled Related Protein 4 | -8.2 |
| 44 | CD146 / MCAM | Cell Adhesion Molecule | -8.2 |
| 45 | COL6A2 | Collagen Type VI Alpha 2 Chain | -8.1 |
| 46 | Vimentin | Vimentin | -7.8 |
| 47 | COL1A1 | Collagen Type I Alpha 1 Chain | -7.7 |
| 48 | CAV1 | Caveolin-1 | -7.6 |
| 49 | IL-8 / CXCL8 | Interleukin-8 | -7.5 |
| 50 | PDGF | Platelet-Derived Growth Factor | -7.5 |
| 51 | CCL2 / MCP-1 | C-C Motif Chemokine Ligand 2 | -7.4 |
| 52 | HSF1 | Heat Shock Factor 1 | -7.4 |
| 53 | FGF5 | Fibroblast Growth Factor 5 | -7.2 |
| 54 | IGF-2 | Insulin-Like Growth Factor 2 | -7.1 |
| 55 | FSP1 / S100A4 | Fibroblast-Specific Protein 1 | -7 |
| 56 | FGF2 | Fibroblast Growth Factor 2 | -7 |
| 57 | PDGFRβ | Platelet-Derived Growth Factor Receptor beta | -7 |
| 58 | CXCL12 / SDF-1 | C-X-C Motif Chemokine Ligand 12 | -6.7 |
| 59 | PDPN | Podoplanin | -6.4 |
| 60 | FXYD1 | FXYD Domain Containing Ion Transport Regulator 1 | -5.8 |
